# Supplementary material for: Rapid, robust plasmid verification by de novo assembly of short sequencing reads
Source: Nucleic Acids Res. 2020 Sep 5;48(18):e106. doi: 10.1093/nar/gkaa727 (PMC7544192; doi:10.1093/nar/gkaa727)
Supplement: gkaa727_Supplemental_File [file gkaa727_supplemental_file.pdf]

Supplementary data for:
Rapid, robust plasmid verification by de novo assembly of short sequencing reads

Jenna. E. Gallegos[1], Mark F. Rogers[2], Charlotte Cialek[2,3], Jean Peccoud[1,2]

[1]Department of Chemical & Biological Engineering, Colorado State University
[2]GenoFAB, Inc.
[3]Department of Biochemistry and Molecular Biology, Colorado State University

Data With Reference Sequences

Supplementary Tables 4 through 9 provide the results of running the pipeline on three datasets having reference sequences. We used default pipeline settings for all but one of the datasets: the input reads for the Schlebach dataset were smaller than the pipeline's minimum of 50 bases for trimmed and filtered reads, so we lowered the minimum to 30 in this case. In each table we provide: the total number of read pairs; original read lengths or range of lengths; the number and percent of pairs that remain after filtering; the assembly size (or indication of a failed or fragmented assembly); the proportion of correct (reference) sequence covered by the assembly, and the fidelity of the covered region given as % ID.

CSU Datasets

Below are results for the datasets generated at Colorado State University (CSU) and used to develop the *de novo* assembly pipeline. There are eight distinct plasmid sequences with six to twelve replicates per plasmid. The plasmids are named *Control*, *Signed*, *Mut-Sig*, *Mut-Ecc*, *Alt-Promoter*, *Alt-Promoter-Truncated*, *p190691* and *401734*.

Replicate	# Reads	Length	Filtered	% Filtered	Assembly	% coverage	% ID	Contamination
A01	41,735	35—251	3,442	8.25%	2,521	100%	100%	16%
A02	25,382	35—251	1,948	7.67%	2,521	100%	100%	6%
A03	23,315	35—251	2,163	9.28%	2,521	100%	100%	4%
A04	27,963	35—251	2,406	8.60%	2,521	100%	100%	10%
A05	23,435	35—251	1,876	8.01%	2,521	100%	100%	10%
A06	37,971	35—251	2,341	6.17%	2,521	100%	100%	5%
A07	28,431	35—251	2,418	8.50%	2,521	100%	100%	1%
A08	22,928	35—251	2,176	9.49%	2,521	100%	100%	1%
A09	34,294	35—251	3,819	11.14%	2,521	100%	100%	64%
A10	24,013	35—251	2,555	10.64%	2,521	100%	100%	1%
A11	24,790	35—251	1,934	7.80%	2,521	100%	100%	5%
A12	29,303	35—251	1,982	6.76%	2,521	100%	100%	6%
B01	35,957	35—251	3,177	8.84%	2,521	100%	100%	39%
B02	25,208	35—251	2,146	8.51%	2,521	100%	100%	3%
B03	28,692	35—251	2,646	9.22%	2,521	100%	100%	5%
B04	39,666	35—251	3,474	8.76%	2,521	100%	100%	3%
B05	30,255	35—251	3,180	10.51%	2,521	100%	100%	9%
B06	21,396	35—251	1,617	7.56%	2,521	100%	100%	10%
B07	23,162	35—251	1,439	6.21%	2,521	100%	100%	6%
B08	70,295	35—251	5,894	8.38%	2,521	100%	100%	72%
B09	20,699	35—251	1,920	9.28%	2,521	100%	100%	93%
B10	22,302	35—251	2,033	9.12%	2,521	100%	100%	1%
B11	24,663	35—251	2,303	9.34%	2,521	100%	100%	3%
B12	30,326	35—251	2,033	6.70%	2,521	100%	100%	3%

Supplementary Table 1: **CSU (datasets A and B)**: the correct sequence, the *Control* plasmid, has 2,521 bases. The pipeline successfully generated the correct sequence from all 24 replicates. Generally the estimated contamination was low, but in three cases (A09, B08 and B09) the estimate was over 64%. However any contamination in the reads did not adversely impact these assemblies.

Replicate	# Reads	Length	Filtered	% Filtered	Assembly	% coverage	% ID	Contamination
C01	27,713	44—251	2,442	8.81%	3,129	100%	100%	16%
C02	34,475	37—251	2,402	6.97%	3,129	100%	100%	15%
C03	23,658	38—251	1,597	6.75%	3,129	100%	100%	1%
C04	21,962	35—251	2,205	10.04%	3,129	100%	100%	1%
C05	18,311	35—251	1,504	8.21%	3,129	100%	100%	1%
C06	24,964	35—251	2,780	11.14%	3,129	100%	100%	1%
C07	23,357	35—251	1,767	7.57%	3,129	100%	100%	3%
C08	23,091	35—251	1,752	7.59%	3,129	100%	100%	3%
C09	29,032	38—251	1,551	5.34%	3,129	100%	100%	1%
C10	32,703	39—251	2,535	7.75%	3,129	100%	100%	2%
C11	20,390	40—251	2,045	10.03%	3,129	100%	100%	20%
C12	28,489	35—251	2,666	9.36%	3,129	100%	100%	1%
D01	36,072	35—251	2,210	6.13%	3,129	100%	100%	22%
D02	28,650	35—251	1,992	6.95%	3,129	100%	100%	2%
D03	28,494	35—251	1,874	6.58%	3,129	100%	100%	1%
D04	27,590	35—251	1,500	5.44%	3,129	100%	100%	9%
D05	47,833	35—251	1,303	2.72%	3,129	100%	100%	98%
D06	27,112	40—251	3,010	11.10%	3,129	100%	100%	9%
D07	25,396	40—251	2,109	8.30%	3,129	100%	100%	4%
D08	27,088	35—251	2,938	10.85%	3,129	100%	100%	23%
D09	26,914	38—251	2,389	8.88%	3,129	100%	100%	6%
D10	22,758	38—251	1,586	6.97%	3,129	100%	100%	23%
D11	19,945	38—251	841	4.22%	3,129	100%	100%	7%
D12	24,451	43—251	1,821	7.45%	3,129	100%	100%	28%
E01	29,289	35—251	2,629	8.98%	3,129	100%	100%	29%
E02	25,529	40—251	2,444	9.57%	3,129	100%	100%	7%
E03	50,723	38—251	5,454	10.75%	3,129	100%	100%	80%
E04	19,295	35—251	1,312	6.80%	3,129	100%	100%	1%
E05	36,623	35—251	2,781	7.59%	3,129	100%	100%	4%
E06	22,549	36—251	1,201	5.33%	3,129	100%	100%	2%
E07	25,757	40—251	2,418	9.39%	3,129	100%	100%	11%
E08	26,171	35—251	2,788	10.65%	3,129	100%	100%	5%
E09	26,378	35—251	2,385	9.04%	3,129	100%	100%	1%
E10	24,406	35—251	2,737	11.21%	3,129	100%	100%	38%
E11	23,163	35—251	1,779	7.68%	3,129	100%	100%	1%
E12	42,024	35—251	2,467	5.87%	3,129	100%	100%	1%
F01	28,845	38—251	1,811	6.28%	3,129	100%	100%	3%
F02	26,446	35—251	2,464	9.32%	3,129	100%	100%	9%
F03	27,246	35—251	2,370	8.70%	3,129	100%	100%	1%
F04	22,013	38—251	1,494	6.79%	3,129	100%	100%	1%
F05	23,993	35—251	2,495	10.40%	3,129	100%	100%	1%
F06	25,152	36—251	1,928	7.67%	3,129	100%	100%	2%
F07	29,965	40—251	1,882	6.28%	3,129	100%	100%	1%
F08	24,182	35—251	1,580	6.53%	3,129	100%	100%	2%
F09	27,051	35—251	2,291	8.47%	3,129	100%	100%	1%
F10	19,607	35—251	2,028	10.34%	3,129	100%	100%	7%
F11	25,384	36—251	1,958	7.71%	3,129	100%	100%	11%
F12	41,362	35—251	2,512	6.07%	3,129	100%	100%	15%

Supplementary Table 2: **CSU datasets C, D, E and F**: all four plasmids (*Signed*, *Mut-Sig*, *Mut-Ecc*, *Alt-Promoter* and *Alt-Promoter-Truncated*, respectively) have a sequence length of 3,129 bases. The pipeline successfully generated the correct sequence from all 48 replicates. Estimated contamination was low except for two cases (D05 and E03) where the estimates were over 80%. However the contamination detected in the reads did not adversely impact these assemblies.

Replicate	# Reads	Length	Filtered	% Filtered	Assembly	% coverage	% ID	Contamination
G01	60,720	35—251	5,969	9.83%	3,293	100%	99.94%	72%
G02	44,789	35—251	3,245	7.25%	3,294	100%	100%	2%
G03	38,851	39—251	3,132	8.06%	3,294	100%	99.97%	1%
G04	35,202	35—251	3,227	9.17%	3,292	100%	99.91%	5%
G05	25,085	35—251	1,607	6.41%	3,294	100%	100%	15%
G06	36,007	39—251	3,005	8.35%	3,293	100%	99.94%	4%
G07	30,358	35—251	2,146	7.07%	3,294	100%	99.97%	1%
G08	31,802	42—251	2,035	6.40%	3,294	100%	99.94%	1%
G09	83,340	35—251	5,236	6.28%	3,294	100%	100%	63%
G10	42,995	35—251	3,214	7.48%	3,295	100%	99.88%	6%
G11	48,778	38—251	4,222	8.66%	3,292	100%	99.94%	7%
G12	46,389	35—251	3,517	7.58%	3,294	100%	100%	6%
H01	35,853	35—251	3,385	9.44%	3,170	100%	100%	3%
H02	22,102	49—251	1,234	5.58%	3,170	100%	99.97%	2%
H03	18,858	35—251	1,401	7.43%	3,169	100%	99.94%	54%
H04	56,629	35—251	3,644	6.43%	3,170	100%	99.97%	49%
H05	39,865	40—251	3,025	7.59%	3,170	100%	100%	39%
H06	25,316	35—251	2,302	9.09%	3,171	100%	99.97%	2%
H07	27,299	39—251	1,970	7.22%	3,170	100%	100%	1%
H08	41,850	35—251	4,347	10.39%	3,170	100%	99.97%	1%
H09	22,401	35—251	1,172	5.23%	3,170	100%	100%	1%
H10	25,202	42—251	2,022	8.02%	3,170	100%	99.94%	47%
H11	27,853	35—251	2,471	8.87%	3,170	100%	100%	1%
H12	36,222	35—251	2,725	7.52%	3,170	100%	99.97%	9%

Supplementary Table 3: **CSU datasets G and H**: the plasmid sequences have 3,294 bases (G, *p190691*) and 3,170 bases (H, *401734*). These plasmids were assembled via Gibson sampling and thus may have insertions or deletions relative to the reference sequence. For this reason, coverage statistics are the result of sequence alignment that allows for gaps in either sequence. These are reflected in the sequence identity that ranges from 99.91% for three indels, to 100% for a perfect match with no insertions or deletions. Three of the 24 assemblies yield contamination estimates above 50% (G01, G09 and H03): for G09 it did not impact the assembly; for G01 and H03 the impact is less clear, as assembly mismatches could arise from either indels or contamination.

Chiniquy Dataset

Replicate	Read pairs	Length	Filtered	% Filtered	Assembly	% Coverage	% ID
1	18,651	32–151	1,530	8.20%	6,588	99.94%	100%
2	17,019	32–151	1,326	7.79%	6,588	99.94%	100%
3	18,734	32–151	1,530	8.16%	6,588	99.94%	100%
4	19,652	32–151	1,386	7.05%	6,588	99.94%	100%
5	19,006	32–151	1,231	6.48%	6,588	99.94%	100%
6	13,484	32–151	903	6.69%	6,588	99.94%	100%
7	21,160	32–151	1,598	7.55%	6,588	99.94%	100%
8	27,082	32–151	1,957	7.23%	6,588	99.94%	100%
9	21,961	32–151	1,399	6.37%	6,588	99.94%	100%
10	25,851	32–151	2,208	8.54%	6,588	99.94%	100%
11	25,012	32–151	1,772	7.08%	6,588	99.94%	100%
12	24,570	32–151	1,682	6.84%	6,588	99.94%	100%
13	21,052	32–151	1,654	7.86%	6,588	99.94%	100%
14	22,490	32–151	1,556	6.92%	6,588	99.94%	100%
15	17,724	32–151	1,072	6.04%	6,588	99.94%	100%
16	23,761	32–151	1,960	8.25%	6,588	99.94%	100%
17	19,576	32–151	1,736	8.87%	6,588	99.94%	100%
18	22,393	32–151	1,928	8.61%	6,588	99.94%	100%
19	20,265	32–151	1,697	8.37%	6,588	99.94%	100%
20	21,516	32–151	1,711	7.95%	6,588	99.94%	100%
21	22,251	32–151	1,625	7.30%	6,588	99.94%	100%
22	19,734	32–151	1,757	8.90%	6,588	99.94%	100%
23	22,742	32–151	1,801	7.92%	6,588	99.94%	100%
24	22,590	32–151	1,814	8.03%	6,588	99.94%	100%

Supplementary Table 4: **Chiniquy plasmid pXMJ19**: 24 sets of sequencing data from [1]. The correct sequence has 6,592 bases. Using default settings, the pipeline consistently generated assemblies from the available FASTQ data. In all cases, the resulting assembly was exactly four bases shy of the correct length. Interestingly, the reference sequence starts and ends with an `AATT` tetramer, making a circular assembly extremely challenging.

Replicate	Read pairs	Length	Filtered	% Filtered	Assembly	% Coverage	% ID
1	6,754	32–151	702	10.39%	4,052	100.34%	99.56%
2	9,912	32–151	1,169	11.79%	4,101	101.56%	98.36%
3	8,698	32–151	777	8.93%	4,040	100.04%	99.85%
4	9,886	32–151	943	9.54%	4,040	100.04%	99.85%
5	9,820	32–151	1,108	11.28%	4,040	100.04%	99.85%
6	9,275	35–151	856	9.23%	4,060	100.54%	99.31%
7	7,188	32–151	760	10.57%	3,923	97.15%	99.72%
8	9,031	35–151	989	10.95%	4,040	100.04%	99.85%
9	9,889	32–151	1,084	10.96%	4,039	100.02%	99.70%
10	10,753	32–151	1,189	11.06%	4,040	100.04%	99.85%
11	8,036	32–151	873	10.86%	4,040	100.04%	99.85%
12	6,933	32–151	654	9.43%	4,040	100.04%	99.83%
13	7,314	32–151	650	8.89%	4,039	100.02%	99.70%
14	5,764	38–151	552	9.58%	3,256	80.63%	99.82%
15	8,740	32–151	875	10.01%	4,033	99.87%	99.73%
16	8,373	32–151	879	10.50%	4,040	100.04%	99.85%
17	9,897	32–151	890	8.99%	4,040	100.04%	99.85%
18	7,961	35–151	860	10.80%	4,048	100.24%	99.65%
19	7,444	32–151	672	9.03%	4,040	100.04%	99.85%
20	8,549	32–151	837	9.79%	4,040	100.04%	99.85%
21	9,657	32–151	1,091	11.30%	4,040	100.04%	99.85%
22	8,438	32–151	767	9.09%	4,030	99.80%	99.73%
23	6,694	32–151	707	10.56%	4,043	100.12%	99.73%
24	7,328	32–151	861	11.75%	4,040	100.04%	99.85%

Supplementary Table 5: **Chiniquy plasmid pms6126**: 24 sets of sequencing data from [1]. The correct sequence has 4,038 bases. Using default settings, the pipeline successfully generated assemblies for all 24 samples. Of these, 20 were slightly longer than the correct sequence (up to 1.6% longer) while four were shorter. All assemblies yielded high fidelity, with 99.31% to 99.85% ID compared with the correct sequence.

Replicate	Read pairs	Length	Filtered	% Filtered	Assembly	% Coverage	% ID
1	23,251	32–151	1,835	7.89%	9,207	88.8%	100.0%
2	23,201	32–151	2,008	8.65%	10,168	98.1%	99.99%
3	18,123	32–151	1,405	7.75%	9,228	89.0%	100.0%
4	22,550	32–151	1,682	7.46%	*fragmented*	—	—
5	23,038	32–151	2,025	8.79%	10,119	97.6%	99.99%
6	23,454	32–151	1,680	7.16%	10,131	97.7%	100.0%
7	17,325	32–151	1,345	7.76%	*fragmented*	—	—
8	19,597	32–151	1,624	8.29%	*fragmented*	—	—
9	15,915	32–151	998	6.27%	*fragmented*	—	—
10	18,983	32–151	1,211	6.38%	*fragmented*	—	—
11	15,682	32–151	981	6.26%	*fragmented*	—	—
12	17,350	32–151	933	5.38%	*fragmented*	—	—
13	18,369	32–151	938	5.11%	*fragmented*	—	—
14	13,103	32–151	723	5.52%	*fragmented*	—	—
15	17,623	32–151	991	5.62%	*fragmented*	—	—
16	17,848	32–151	922	5.17%	*fragmented*	—	—
17	17,733	32–151	963	5.43%	*fragmented*	—	—
18	19,919	32–151	1,248	6.27%	*fragmented*	—	—
19	17,990	32–151	963	5.35%	*fragmented*	—	—
20	14,615	32–151	795	5.44%	*fragmented*	—	—
21	19,897	32–151	1,233	6.20%	9,346	90.1%	99.99%
22	18,896	32–151	1,029	5.45%	*fragmented*	—	—
23	15,960	32–151	752	4.71%	*fragmented*	—	—
24	15,614	32–151	933	5.98%	*fragmented*	—	—

Supplementary Table 6: **Chiniquy plasmid pGEN-292**: 24 sets of sequencing data from [1]. The correct sequence has 10,370 bases. Using default settings, the pipeline was able to generate complete assemblies for six of the 24 samples. For the six successful assemblies, coverage ranged from 88.8% to 98.1% with sequence identity close to 100%. Using the `repeat-match` tool from MuMMer [2], we find six repeats in the reference sequence ranging up to 38 bases in length, longer than the smallest input reads. Using parameter manipulation to relax filtering requirements and increase the number of input reads, we were able to boost the number of successful assemblies to 12 out of 24.

Replicate	Read pairs	Length	Filtered	% Filtered	Assembly	% Coverage	% ID
1	10,767	32–151	702	6.52%	*fragmented*	—	—
2	13,153	32–151	1,169	8.88%	5,395	89.6%	99.87%
3	8,520	42–151	777	9.12%	2,293	38.1%	99.87%
4	9,052	32–151	943	10.42%	3,114	51.7%	99.84%
5	5,484	43–151	1,108	20.24%	*Failed*	—	—
6	9,959	45–151	856	8.59%	*fragmented*	—	—
7	10,597	32–151	760	7.17%	*fragmented*	—	—
8	10,690	32–151	989	9.25%	4,212	69.9%	99.83%
9	9,509	34–151	1,084	11.40%	3,206	53.2%	99.81%
10	10,568	36–151	1,189	11.26%	*fragmented*	—	—
11	10,040	37–151	873	8.69%	3,831	63.6%	99.87%
12	11,950	34–151	654	5.47%	*fragmented*	—	—
13	11,211	44–151	650	5.80%	*fragmented*	—	—
14	11,241	41–151	552	4.91%	*fragmented*	—	—
15	9,501	36–151	875	9.21%	3,279	54.5%	99.82%
16	10,293	36–151	879	8.54%	*fragmented*	—	—
17	9,075	37–151	890	9.81%	*fragmented*	—	—
18	10,218	32–151	860	8.41%	2,752	45.7%	99.78%
19	9,478	38–151	672	7.09%	*fragmented*	—	—
20	9,294	41–151	837	9.00%	*fragmented*	—	—
21	8,397	42–151	1,091	13.00%	2,913	48.4%	99.76%
22	6,425	40–151	767	11.94%	2,810	46.7%	99.86%
23	8,353	35–151	707	8.46%	2,555	42.4%	99.77%
24	9,621	36–151	861	8.95%	2,019	33.5%	99.70%

Supplementary Table 7: **Chiniquy plasmid pskb3-CopR1598**: 24 sets of sequencing data from [1]. The correct sequence has 6,029 bases. Using default settings, the pipeline generated assemblies successfully for 12 of the 24 replicates. Coverage ranged from 33.5% to 89.6%, while fidelity ranged from 99.76% to 99.87%. As with the pGEN-292 example, using parameter manipulation to relax filtering requirements and increase the number of input reads, we were able to boost the number of successful assemblies to 19 out of 24 and eliminate the failed assembly for replicate 5.

Schlebach Dataset

Sample	Read pairs	Length	Filtered	% Filtered	Assembly	Coverage	%ID
GSF2504-CFTR2-C1_S43	658,594	78	375,162	56.96%	9,477	99.93%	99.68%
GSF2504-CFTR2-C2_S44	706,345	78	389,765	55.18%	9,477	99.93%	99.68%
GSF2509-CFTR2-C3_S16	322,411	42	154,560	47.94%	9,476	99.92%	99.68%
GSF2509-CFTR2-C4_S17	271,536	42	127,876	47.09%	9,477	99.93%	99.67%
GSF2509-CFTR2-C5_S18	249,821	42	119,919	48.00%	9,477	99.93%	99.67%
GSF2509-CFTR2-C6_S19	265,136	42	127,563	48.11%	9,476	99.92%	99.66%
GSF2509-CFTR2-C7_S20	261,106	42	124,740	47.77%	9,477	99.93%	99.68%
GSF2509-CFTR2-C8_S21	342,932	42	164,293	47.91%	9,477	99.93%	99.67%
GSF2509-CFTR2-C9_S22	309,151	42	148,473	48.03%	9,477	99.93%	99.68%
GSF2509-CFTR2-C10_S23	318,602	42	156,002	48.96%	9,477	99.93%	99.68%
GSF2509-CFTR2-C11_S24	354,237	42	174,109	49.15%	9,477	99.93%	99.68%
GSF2509-CFTR2-C12_S25	315,275	42	153,117	48.57%	9,476	99.92%	99.68%
GSF2509-CFTR2-C13_S26	342,410	42	165,623	48.37%	9,477	99.93%	99.68%
GSF2509-CFTR2-C15_S27	293,907	42	146,925	49.99%	9,477	99.93%	99.68%
GSF2509-CFTR2-C16_S28	301,952	42	141,557	46.88%	9,477	99.93%	99.68%
GSF2509-CFTR2-C18_S29	345,954	42	163,757	47.33%	9,477	99.93%	99.68%
GSF2509-CFTR2-C19_S30	418,753	42	193,085	46.11%	9,477	99.93%	99.67%
GSF2509-CFTR2-C20_S31	340,930	42	157,735	46.27%	9,477	99.93%	99.68%
GSF2509-CFTR2-C21_S32	319,423	42	149,306	46.74%	9,477	99.93%	99.68%
GSF2509-CFTR2-C22_S33	308,373	42	141,013	45.73%	9,476	99.92%	99.68%
GSF2509-CFTR2-C23_S34	307,857	42	142,928	46.43%	9,477	99.93%	99.68%
GSF2509-CFTR2-C24_S35	350,680	42	164,608	46.94%	9,477	99.93%	99.66%

Supplementary Table 8: **Schlebach plasmid data**: the Schlebach lab at Indiana University provided us with 24 sets of paired-end reads drawn from a sequence 9,484 bases in length. These data presented two challenges: first, the reference sequence includes a string of ten `N` bases, so is not completely defined. Second, read lengths were substantially shorter than those used to develop the pipeline, ranging from just 42 to 78 bases in length. Assembly coverage is consistently over 99.9% of the full sequence length, while fidelity is consistently just under 99.7% ID.

Pasin Dataset

Sample	Read pairs	Length	Filtered	% Filtered	Assembly	Coverage	%ID
Wasabi mottle virus	36,003	142	24,172	67.14%	*fragmented*	—	—
Turnip mosaic virus	74,743	142	47,862	64.04%	14,281	99.0%	100%
Turnip curly top virus	82,280	142	53,139	64.58%	*fragmented*	—	—
Cauliflower mosaic virus	75,337	142	50,723	67.33%	*fragmented*	—	—

Supplementary Table 9: **Cauliflower mosaic virus data**: Illumina MiSeq data from a 2018 study [3] provide four sets of reads for four distinct species of varying lengths. Running the pipeline with default settings yielded just one complete assembly with a length over 14KB, much larger than any of the references. The only close match is with the Turnip mosaic virus, which is extremely close at 99% coverage and 100% ID. It is worth noting that it is the only sequence that does not contain long repeats: with `repeat-match` we find that the other sequences include long repeats of 222 to 759 bases, longer than any of the input reads. In addition, data quality scores were significantly higher than for our own data, so filtered reads retained more than 60% of the original reads.

Data Without Reference Sequences

Supplementary Tables 10 through 11 provide the results of running the pipeline with default settings on a dataset without any reference sequences. In each table we provide the same columns as above, but lacking any reference sequence, we replace % ID with the estimated likelihood of contamination for each sample.

SeqWell Dataset

Sample	Read pairs	Length	Filtered	% Filtered	Assembly	Contamination
A01	73,248	35—251	6,708	9.16%	6,505	1%
A02	59,992	35—251	6,723	11.21%	6,141	1%
A03	41,589	35—251	4,006	9.63%	*fragmented*	-
A04	47,506	35—251	5,385	11.34%	*fragmented*	-
A05	50,753	35—251	4,862	9.58%	7,160	1%
A06	70,691	35—251	5,828	8.24%	7,160	1%
A07	43,123	37—251	5,123	11.88%	5,057	5%
A08	61,290	35—251	6,413	10.46%	4,327	1%
A09	50,804	35—251	5,743	11.30%	4,327	13%
A10	53,864	35—251	7,054	13.10%	12,364	1%
A11	46,636	35—251	4,145	8.89%	6,209	1%
A12	39,933	35—251	3,472	8.69%	7,105	1%
B01	52,532	35—251	5,824	11.09%	5,971	1%
B02	43,912	35—251	5,343	12.17%	11,490	1%
B03	36,538	35—251	4,269	11.68%	*fragmented*	-
B04	45,409	35—251	5,859	12.90%	*fragmented*	-
B05	45,130	35—251	6,022	13.34%	*fragmented*	-
B06	35,888	35—251	4,121	11.48%	*fragmented*	-
B07	49,294	35—251	5,020	10.18%	*fragmented*	-
B08	75,021	35—251	9,257	12.34%	*fragmented*	-
B09	33,220	40—251	4,185	12.60%	10,251	1%
B10	38,254	35—251	3,990	10.43%	7,247	6%
B11	54,145	38—251	5,275	9.74%	10,174	1%
B12	48,085	35—251	3,300	6.86%	10,111	1%
C01	33,169	35—251	3,570	10.76%	10,125	1%
C02	47,149	35—251	3,741	7.93%	6,743	1%
C03	37,834	35—251	4,618	12.21%	*fragmented*	-
C04	36,276	35—251	4,581	12.63%	10,200	1%
C05	41,831	35—251	4,356	10.41%	6,792	1%
C06	35,783	35—251	5,472	15.29%	7,855	1%
C07	40,229	35—251	4,493	11.17%	9,748	1%
C08	49,017	35—251	6,348	12.95%	*fragmented*	-
C09	50,447	35—251	4,541	9.00%	*fragmented*	-
C10	27,270	37—251	3,144	11.53%	*fragmented*	-
C11	36,137	38—251	5,144	14.23%	*fragmented*	-
C12	46,135	35—251	4,596	9.96%	4,592	1%
D01	52,930	37—251	6,317	11.93%	*fragmented*	-
D02	40,510	35—251	4,013	9.91%	14,436	1%
D03	51,146	35—251	5,301	10.36%	8,193	1%
D04	41,612	35—251	3,449	8.29%	*fragmented*	-
D05	48,676	35—251	3,822	7.85%	8,066	92%
D06	8,733	35—251	1,293	14.81%	6,427	2%
D07	49,778	35—251	6,000	12.05%	*fragmented*	-
D08	50,173	35—251	5,968	11.89%	*fragmented*	-
D09	38,945	35—251	3,403	8.74%	*fragmented*	-
D10	17,807	35—251	2,242	12.59%	4,016	100%
D11	62,227	35—251	4,424	7.11%	7,784	23%
D12	75,119	36—251	8,568	11.41%	4,834	1%

Supplementary Table 10: **SeqWell data, samples A-D**: results from running the pipeline using default parameters. Of these 48 samples, 28 yielded reliable assemblies, two yielded strong evidence of contamination (estimated probability 92% to 100%) and 18 yielded fragmented assemblies, also suggesting contamination.

Sample	Read pairs	Length	Filtered	% Filtered	Assembly	Contamination
E01	17,111	35—251	2,273	13.28%	4,145	98%
E02	20,790	43—251	2,892	13.91%	*fragmented*	-
E03	54,081	35—251	7,713	14.26%	4,459	5%
E08	30,242	39—251	5,095	16.85%	*fragmented*	-
E09	37,923	35—251	5,136	13.54%	*fragmented*	-
E10	34,462	41—251	6,049	17.55%	*fragmented*	-
E11	37,640	38—251	5,323	14.14%	*fragmented*	-
E12	57,021	40—251	6,708	11.76%	*fragmented*	-
F01	23,290	36—251	2,375	10.20%	*fragmented*	-
F02	32,598	35—251	4,792	14.70%	6,631	1%
F03	32,523	35—251	5,922	18.21%	12,621	1%
F04	25,851	38—251	2,852	11.03%	5,928	66%
F05	37,153	35—251	4,556	12.26%	4,689	1%
F06	12,301	35—251	1,780	14.47%	5,085	100%
F07	39,560	35—251	3,645	9.21%	6,938	1%
F08	64,804	35—251	7,280	11.23%	4,439	1%
F09	45,563	37—251	4,394	9.64%	6,244	1%
F10	31,273	35—251	3,980	12.73%	7,429	1%
F11	24,348	40—251	2,393	9.83%	6,253	90%
F12	25,134	35—251	2,072	8.24%	7,713	91%
G01	32,194	41—251	4,625	14.37%	9,858	15%
G02	48,396	35—251	5,437	11.23%	13,452	1%
G03	31,234	35—251	3,600	11.53%	6,926	2%
G04	19,012	38—251	2,464	12.96%	4,162	100%
G05	38,392	42—251	4,541	11.83%	8,493	1%
G06	54,934	35—251	6,836	12.44%	7,751	1%
G07	38,738	36—251	3,535	9.13%	8,984	1%
G08	41,833	35—251	4,263	10.19%	8,865	2%
G09	56,501	35—251	6,201	10.98%	8,450	99%
G10	50,123	40—251	4,921	9.82%	8,510	1%
G11	32,229	37—251	4,341	13.47%	5,705	80%
G12	34,989	42—251	4,581	13.09%	6,064	56%
H01	20,313	36—251	2,980	14.67%	4,034	96%
H02	18,640	44—251	2,026	10.87%	5,668	51%
H03	36,518	35—251	3,446	9.44%	*fragmented*	-
H04	27,884	37—251	4,118	14.77%	*fragmented*	-
H06	51,768	35—251	6,140	11.86%	*fragmented*	-
H07	46,268	35—251	5,396	11.66%	*fragmented*	-
H08	55,197	35—251	4,838	8.76%	8,671	1%
H09	27,286	42—251	1,716	6.29%	8,187	1%
H10	21,113	35—251	2,592	12.28%	*fragmented*	-
H11	18,594	42—251	2,330	12.53%	9,029	100%
H12	21,266	35—251	2,318	10.90%	*fragmented*	-

Supplementary Table 11: **SeqWell data, samples E-H**: results from running the pipeline using default parameters. Of these 43 samples, 18 yield reliable assemblies, 12 yield evidence of contamination and 13 yield fragmented assemblies.

Software

The pipeline scripts and sample data are available at the following URL: https://bitbucket.org/genofabinc/oss/src/master/denovo

References

[1] J. Chiniquy, M. Garber, A. Mukhopadhyay, and N. Hillson. Fluorescent amplification for next generation sequencing (FA-NGS) library preparation. *BMC Genomics*, 21(1):1–9, 2020.

[2] Arthur L Delcher, Adam Phillippy, Jane Carlton, and Steven L Salzberg. Fast algorithms for large-scale genome alignment and comparison. *Nucleic Acids Research*, 30(11):2478–2483, 2002.

[3] Fabio Pasin, Xuan-An Tseng, Leonor C Bedoya, Jahangir Heydarnejad, Ting-Chin Deng, Juan Antonio García, and Yet-Ran Chen. Streamlined generation of plant virus infectious clones using the pLX mini binary vectors. *Journal of Virological Methods*, 262:48–55, 2018.
